# Identification of Non-Coding RNAs Associated with Telomeres Using a Combination of enChIP and RNA Sequencing

**DOI:** 10.1371/journal.pone.0123387

**Published:** 2015-04-13

**Authors:** Toshitsugu Fujita, Miyuki Yuno, Daisuke Okuzaki, Rieko Ohki, Hodaka Fujii

**Affiliations:** 1 Chromatin Biochemistry Research Group, Combined Program on Microbiology and Immunology, Research Institute for Microbial Diseases, Osaka University, Suita, Osaka, Japan; 2 DNA-chip Development Center for Infectious Diseases, Research Institute for Microbial Diseases, Osaka University, Suita, Osaka, Japan; 3 Division of Rare Cancer Research, National Cancer Center Research Institute, Tokyo, Japan; University of Crete, GREECE

## Abstract

Accumulating evidence suggests that RNAs interacting with genomic regions play important roles in the regulation of genome functions, including X chromosome inactivation and gene expression. However, to our knowledge, no non-biased methods of identifying RNAs that interact with a specific genomic region have been reported. Here, we used enChIP-RNA-Seq, a combination of engineered DNA-binding molecule-mediated chromatin immunoprecipitation (enChIP) and RNA sequencing (RNA-Seq), to perform a non-biased search for RNAs interacting with telomeres. In enChIP-RNA-Seq, the target genomic regions are captured using an engineered DNA-binding molecule such as a transcription activator-like protein. Subsequently, RNAs that interact with the target genomic regions are purified and sequenced. The RNAs detected by enChIP-RNA-Seq contained known telomere-binding RNAs, including the telomerase RNA component (*Terc*), the RNA component of mitochondrial RNA processing endoribonuclease (*Rmrp*), and Cajal body-specific RNAs. In addition, a number of novel telomere-binding non-coding RNAs were also identified. Binding of two candidate non-coding RNAs to telomeres was confirmed by immunofluorescence microscopy and RNA fluorescence *in situ* hybridization (RNA-FISH) analyses. The novel telomere-binding non-coding RNAs identified here may play important roles in telomere functions. To our knowledge, this study is the first non-biased identification of RNAs associated with specific genomic regions. The results presented here suggest that enChIP-RNA-Seq analyses are useful for the identification of RNAs interacting with specific genomic regions, and may help to contribute to current understanding of the regulation of genome functions.

## Introduction

Accumulating evidence suggests that RNAs interact with genomic regions to regulate their functions [1]. The regulation of genome functions by interacting RNAs occurs during X chromosome inactivation [2, 3], genomic imprinting [3], transcriptional regulation [4], and other processes. The interaction of a specific RNA with genomic regions can be detected by several techniques, such as fluorescent *in situ* hybridization (FISH) [5] and oligonucleotide-mediated affinity purification [6–9]. These methods can be used if information on the candidate RNAs is available; however, they are not suitable for non-biased searches for RNAs that interact with specific genomic regions.

Recently, we developed two locus-specific chromatin immunoprecipitation (locus-specific ChIP) technologies, namely insertional ChIP (iChIP) [10–15] and engineered DNA-binding molecule-mediated ChIP (enChIP) [16–18]. These locus-specific ChIP technologies enable the purification of specific genomic regions while retaining molecular interactions, and the combination of iChIP or enChIP with mass spectrometry can identify proteins that interact with specific genomic regions [11, 15–18]. We also showed that the combination of iChIP or enChIP with RT-PCR can detect RNA species that interact with the chicken β-globin HS4 element [11], telomeres [17], and the chicken *Pax5* locus [14].

Telomeres are specialized chromatin structures that protect the ends of chromosomes from being recognized as broken DNA [19]. Telomeres consist of a 5–15 kb tandem repetitive array of T_2_AG_3_ sequences (telomeric repeats) and interacting RNAs and proteins, which form a large DNA-RNA-protein complex. Telomeric repeats are maintained by the action of telomerase, which comprises a protein component named telomerase reverse transcriptase (TERT), and an RNA component named *Terc* [20, 21]. In addition to *Terc*, other RNAs also interact with the telomerase complex via a direct interaction with TERT or an indirect interaction with other telomerase-associated proteins [22, 23]. Although extensive analyses have been performed to identify RNAs associated with telomeres, current knowledge of telomere-associated non-coding RNAs is far from complete.

Here, We Combined Enchip With Rna Sequencing (Rna-Seq), Hereafter Referred To As Enchip-Rna-Seq, To Perform A Non-Biased Investigation Of Non-Coding Rnas That Interact With Telomeres. Using This Method, Both Known And Novel Telomere-Interacting Rnas Were Identified. We Propose That Enchip-Rna-Seq Is A Useful Tool For Analyzing The Effects Of Specific Rnas On Genome Functions.

## Results and Discussion

### Identification of RNA species associated with telomeres by enChIP-RNA-Seq

We recently reported the isolation of telomeres by enChIP using a transcription activator-like (TAL) protein that recognizes telomeres (Tel-TAL) [17]. In this previous study, Tel-TAL fused with a 3xFLAG-tag and nuclear localization signal (NLS), hereafter referred as 3xFN-Tel-TAL, was expressed in the Ba/F3 mouse hematopoietic cell line [24], which expresses functional telomerase [25]. The cells were treated with formaldehyde and the crosslinked chromatin was fragmented by sonication. Next, chromatin complexes bound to 3xFN-Tel-TAL were immunoprecipitated with an anti-FLAG antibody (Ab), and telomere-binding proteins and *Terc* were identified by mass spectrometry and RT-PCR analyses, respectively [17].

**Fig 1:**
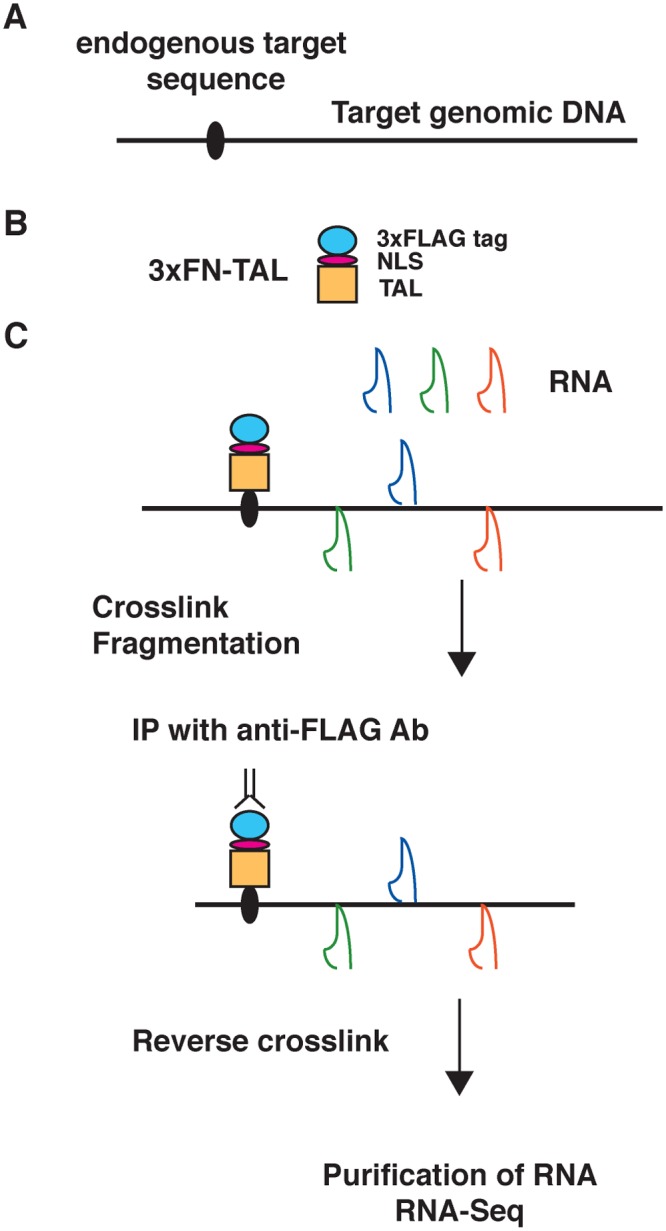
Overview of the enChIP-RNA-Seq analysis. (**A, B**) The enChIP-RNA-Seq system comprises a fusion molecule consisting of a tag(s), a nuclear localization signal (NLS), and an engineered DNA-binding molecule such as a transcription activator-like (TAL) protein, which recognizes endogenous target DNA sequences. The 3xFN-TAL protein comprising a 3xFLAG-tag, an NLS, and a TAL protein recognizing the target sequence is an example of the fusion molecule. (**C**) The 3xFN-TAL protein is expressed in a cell. If necessary, the sample is crosslinked with formaldehyde or another crosslinker, and then the chromatin is fragmented by sonication, enzymatic digestion, or another method. The chromatin complexes are purified by immunoprecipitation with an anti-FLAG Ab. Subsequently, the crosslinking is reversed (if required) and RNAs are purified and subjected to RNA-Seq.

**Fig 2:**
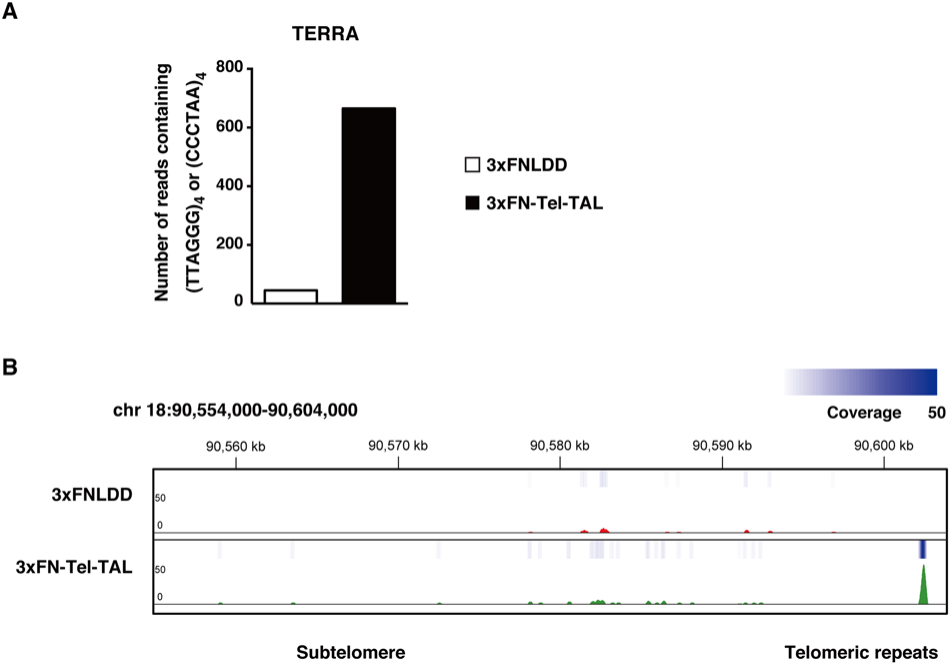
Detection of TERRAs as RNAs associated with telomeres by enChIP-RNA-Seq. (**A**) The numbers of TERRA transcripts in the negative control (3xFNLDD) and 3xFN-Tel-TAL enChIP-RNA-Seq samples. (**B**) Mapping of TERRA transcripts at a telomeric region in chromosome 18.

Here, we used enChIP-RNA-Seq to identify telomere-associated RNAs in a non-biased manner (Fig 1). The RNAs were isolated from Ba/F3 cells expressing 3xFN-Tel-TAL [17] or 3xFLAG-tag-fused LexA (3xFNLDD) as a negative control [17]. We showed previously that 3xFN-Tel-TAL binds specifically to telomeres and enrichment of irrelevant genomic regions is marginal when enChIP is performed using this protein [17]. RNAs that were more abundant in isolates from enChIP from Ba/F3 expressing 3xFN-Tel-TAL than those from Ba/F3 expressing 3xFNLDD were considered as potential telomere-binding RNAs. The non-coding RNAs that potentially interact with telomeres are shown in Table 1 and S1 Table (S2 Table shows the full list of RNAs including both coding and non-coding RNAs that potentially interact with telomeres). The list contains known telomere-binding RNAs, such as *Terc* [20, 21], the RNA component of mitochondrial RNA processing endoribonuclease (*Rmrp*) [23], and small Cajal body-specific RNAs (scaRNAs) [22]. In addition, based on the number of reads containing (TTAGGG)_4_ or (CCCTAA)_4_ motifs [26], we detected a specific enrichment of telomeric repeat-containing RNAs (TERRAs) [27, 28] in the RNA-Seq data from the 3xFN-Tel-TAL samples (Fig 2).

**Table 1:** Examples of RNAs associated with telomeres identified by enChIP-RNA-Seq.

Categories	RNAs
Telomerase components	*Terc*, *Rmrp*
Telomeric RNAs	TERRAs
scaRNAs	*Scarna6*, *Scarna10*, *Scarna13*, *Scarna2*
H/ACA snoRNAs	*Snora23*, *Snora74a*, *Snora73b*, *Snora73a*
C/D snoRNAs	*Snord17*, *Snord15a*, *Snord118*
lncRNA	*Neat1*

As mentioned above, the telomerase holoenzyme consists of TERT, a protein with reverse transcriptase activity, and *Terc*, which serves as a template for the telomere repeats [20] and is a member of the H/ACA family of RNAs [29, 30]. A previous RT-PCR analysis detected *Terc* in telomeres purified by enChIP [17]. *Rmrp* is a non-coding RNA that is found in the nucleolus and mitochondria [31], and is mutated in the inherited pleiotropic syndrome cartilage-hair hypoplasia [32]. TERT and *Rmrp* form a ribonucleoprotein complex that retains RNA-dependent RNA polymerase activity [23]. The scaRNAs, which are also members of the H/ACA RNA family [29, 30], are localized in Cajal bodies and are involved in modifying splicing RNAs. Furthermore, scaRNAs interact with telomere Cajal body protein 1 (TCAB1) at telomeres [22]. TERRAs are UUAGGG repeat-containing RNAs that are transcribed from subtelomeric regions [27] and may play important roles in telomere functions [26–28].

Several members of the H/ACA family of small nucleolar RNAs (snoRNAs) were more abundant in isolates from enChIP from Ba/F3 expressing 3xFN-Tel-TAL than those from Ba/F3 expressing 3xFNLDD (Table 1 and S1 Table). The functional telomerase complex contains dyskerin, an RNA-binding protein that recognizes the H/ACA sequence motif [33, 34]; therefore, it is likely that these H/ACA snoRNAs are recruited to telomeres through interactions with dyskerin [33, 34].

The fact that we detected known telomere-binding RNAs using enChIP-RNA-Seq suggests that it is feasible to use this technology to perform non-biased identification of RNAs interacting with genomic regions of interest *in vivo*. In addition to known telomere-binding RNAs, a number of novel potential telomere-binding RNAs were also identified using enChIP-RNA-Seq, including long non-coding RNAs (lncRNAs) and members of the snoRNA family containing C and D motifs (C/D snoRNAs) (Table 1 and S1 Table). To identify a new functional class of lncRNAs that are enriched in telomeres, we re-analyzed the assembly and quantification of the RNA-Seq data (Table 2 and S3 Table). Overall, the non-coding RNAs identified using enChIP-RNA-Seq might play important roles in telomere biology.

**Table 2:** Telomere-enriched lncRNAs detected by enChIP-RNA-Seq.

Chromosomal location (mm9)	Strand	Length (base)	Covered probe (ID)	Ensembl /NONCODEv3 ID	NONCODEv4 ID	Log2 ratio (telomere / negative control)
chr16:4871450–4874336	Reverse	2,887	A_30_P01027969, A_30_P01023954, A_30_P01020286	n278024, n285321, n288599	NONMMUT030614, NONMMUT030615	3.084
chr2:156213799–156216885	Reverse	3,087	A_30_P01019211, A_30_P01022011, A_30_P01026195	n414189, n263684	NONMMUT040754	2.477
chr18:13114368–13118513	Forward	4,146	A_30_P01017429, A_30_P01031244, A_30_P01017747	N.A.	N.A.	2.430
chrX:90997596–91009570	N.D.	11,975	A_30_P01022594, A_30_P01028927, A_30_P01027891, A_30_P01021513, A_30_P01032697, A_30_P01025876, A_30_P01022054, A_30_P01029616, A_30_P01031912, A_30_P01028449, A_30_P01031411, A_30_P01027235, A_30_P01018764, A_30_P01024096, A_30_P01018317, A_30_P01029941, A_30_P01032299, A_30_P01032782, A_30_P01020940	N.A.	NONMMUT090858	2.283
chrX:91000652–91005855	Reverse	5,204	A_30_P01033041, A_30_P01020922	N.A.	NONMMUT090858	2.145
chrX:11684505–11685304	Forward	800	A_30_P01020753, A_30_P01022354, A_30_P01031956	ENSMUST00000043441, ENSMUST00000145872, ENSMUST00000123004	N.A.	2.145
chrX:18734107–18744367	Reverse	10,261	A_30_P01020672, A_30_P01027310, A_30_P01027623	n422780	NONMMUT089161, NONMMUT089160	2.101
chr6:31233513–31241062	N.D.	7,550	A_30_P01024814, A_30_P01030148, A_30_P01025627, A_30_P01022038	n413169, n421246, n412834, n295685, n412488, n412294, n412483, n416153, n412534, n413041, n413072, n411907, n416150, n416152, n423751, n416151, n421247, n412736, n412401	NONMMUT069481	2.020
chr2:153325671–153352170	N.D.	26,500	A_30_P01021821, A_30_P01031381	ENSMUST00000035346	N.A.	1.463
chr6:83368262–83407280	Reverse	39,019	A_30_P01023363, A_30_P01027012, A_30_P01026288, A_30_P01024889, A_30_P01026790, A_30_P01019688, A_30_P01020253, A_30_P01032016, A_30_P01030413, A_30_P01022314	n268007	NONMMUT071136, NONMMUT071138, NONMMUT071139, NONMMUT071141, NONMMUT071140	1.369

N.A., not available. N.D., not determined.

### Confirmation of localization of the candidate RNAs at telomeres by RNA-FISH

**Fig 3:**
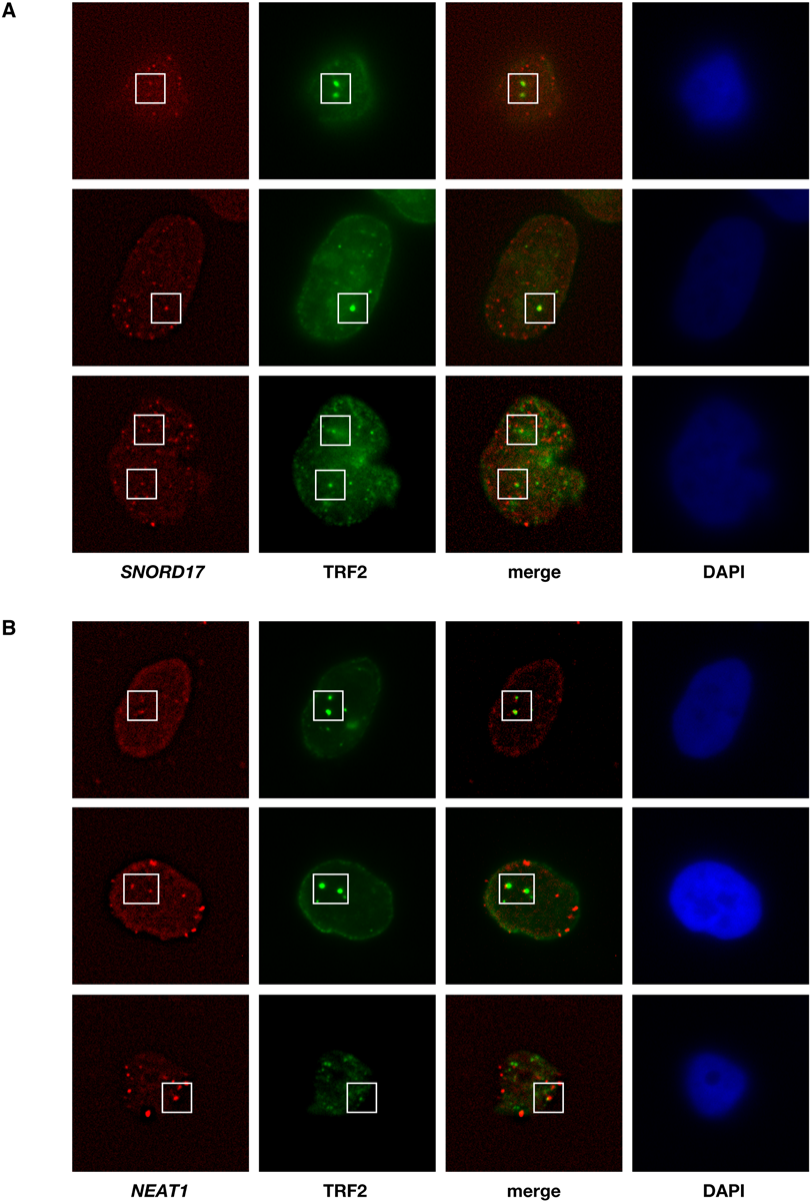
Localization of candidate RNAs at telomeres. Localization of *SNORD17* (**A**) and *NEAT1* (**B**) at telomeres in human U-2 OS cells. Cells were fixed and sequentially incubated with Abs against TRF-2 and AlexaFluor 488-conjugated anti-mouse IgG. Subsequently, the cells were hybridized with the RNA probes and subjected to fluorescence microscopy. Three different cells are shown.

To verify association of the candidate RNAs identified using enChIP-RNA-Seq with telomeres, RNA-FISH analyses were performed using the human osteosarcoma U-2 OS cell line. These experiments showed significant co-localization of two candidate RNAs, namely *SNORD17* (snoRNA, C/D box 17) and *NEAT1* (nuclear-enriched abundant transcript 1), with TRF2, a marker protein of telomeres (Fig 3). These results confirmed the localization of the two candidate RNAs with telomeres. It is of note that not all the RNAs' foci are located at telomeres, suggesting that these RNAs may function not only in telomeres but also in other regions in the nucleus.

*Snord17* belongs to the C/D snoRNA family [30]. Specific enrichment of other members of the C/D snoRNA family, including *Snord15a* and *Snord118*, were also present in telomeres isolated by enChIP (Table 1 and S1 Table). The C/D snoRNAs play important roles in major biological processes, such as translation, mRNA splicing, and genome stability [30]. Although involvement of the H/ACA snoRNAs in telomere biology is well documented [33, 34], to our knowledge, the results presented here are the first to suggest the potential involvement of the C/D snoRNAs in telomere biology. *Neat1* is a lncRNA that is localized to and is essential for the formation of paraspeckles [35]. The enChIP-RNA-Seq data suggest that *Neat1* is also associated with telomeres and may be involved in telomere biology. Consistent with this idea, it has been reported that the localization of *Neat1* is similar to that of TERRAs transcribed from telomeres [36]. It will be interesting to determine how these newly-identified RNAs are involved in telomere functions.

### Non-biased identification of RNAs associated with specific genomic regions by enChIP-RNA-Seq

Here, we used enChIP-RNA-Seq to perform a non-biased search for RNAs interacting with telomeres. This approach can easily be applied to low copy number loci; in fact, we have identified RNAs associated with a single copy gene using RNA-Seq combined with iChIP or enChIP using the CRISPR system (T.F. and H.F., manuscript in preparation). This approach would be a method of choice for identifying RNAs that are associated with a specific genomic locus *in vivo*.

## Conclusions

This Study Describes The Use Of Enchip-Rna-Seq To Detect Known Telomere-Binding Non-Coding Rnas, Including *Terc*, *Rmrp*, Terras, And Scarnas (Table 1 And Fig 2), As Well As A Number Of Novel Potential Telomere-Binding Non-Coding Rnas (Tables 1 And 2). The Localization Of Two Of The Candidate Rnas At Telomeres Was Confirmed By Immunofluorescence Microscopy And Rna-Fish Analyses (Fig 3). The Novel Potential Telomere-Binding Rnas Identified Here May Play Important Roles In Telomere Functions. Our Results Also Suggest That Enchip-Rna-Seq Analyses May Be Useful For The Identification Of Rnas Interacting With Specific Genomic Regions *In Vivo*.

## Materials and Methods

### Cells

The Ba/F3 mouse hematopoietic cell line [24] was obtained from the RIKEN BioResource Center (RCB0805). The generation of Ba/F3 cells stably expressing 3xFNLDD or 3xFN-Tel-TAL has been described previously [17]. The U-2 OS human osteosarcoma cell line [37] was obtained from ATCC (HTB-96). The Ba/F3-derived cells and U-2 OS cells were cultured as described previously [17].

### enChIP-RNA-Seq analysis

Purification of RNAs following enChIP was performed as described previously [17], with some modifications. Briefly, 2 × 10^7^ Ba/F3 cells expressing 3xFNLDD or 3xFN-Tel-TAL were fixed with 1% formaldehyde at 37°C for 5 min. The chromatin fraction was extracted and fragmented by sonication as described previously [11]. The sonicated chromatin was pre-cleared with normal mouse IgG (Santa Cruz Biotechnology) conjugated to Dynabeads-Protein G (Invitrogen), and then incubated with an anti-FLAG M2 Ab (Sigma-Aldrich) conjugated to Dynabeads-Protein G at 4°C. After washing, the total RNA was purified using Isogen II (Nippon Gene) and the Direct-zol RNA MiniPrep Kit (Zymo Research), and treated with DNase I. To obtain a list of RNAs that were differentially present between the two groups (S2 Table), the purified RNAs from cells expressing 3xFNLDD (194.1 ng) or 3xFN-Tel-TAL (160.5 ng) were subjected to RNA-Seq analyses (Takara Bio Inc.). The list of RNAs was sorted according to fold enrichment (read counts of cells expressing 3xFN-Tel-TAL / read counts of cells expressing 3xFNLDD). The non-coding RNAs that were identified as enriched at telomeres (>1.4-fold) are shown in S1 Table. The raw RNA-Seq data have been deposited in the NCBI Gene Expression Omnibus (GEO) database with accession number GSE60425. Details of the sequencing protocol are described on the GEO website.

### Detection of TERRAs in the RNA-Seq data

To estimate the number of putative TERRAs, reads containing (TTAGGG)_4_ or (CCCTAA)_4_ repeats were extracted from each fastq file using the grep command in the UNIX system, as described previously [26].

### Identification of lncRNAs in the RNA-Seq data

To identify a new functional class of lncRNAs enriched in telomeres, the assembly and quantification of the RNA-Seq data were re-analyzed using AvadisNGS software (ver. 1.5.1; Strand Sciences). A total of 3,540 lncRNAs were extracted using Agilent lncRNA probes (Design ID: 028005, Agilent Technologies). The lncRNA probes (16,251 probes, http://www.genomics.agilent.com/article.jsp?crumbAction=push&pageId=1520) were re-annotated according to the NONCODE v3.0 database (http://www.noncode.org/) (S3 Table). The RNA-Seq data were normalized according to the number of reads per million, and the lncRNAs were selected by filtering using cutoffs of more than 50 read counts for the 3xFN-Tel-TAL sample and less than 500 read counts for the control 3xFNLDD sample. A total of 611 lncRNAs passed the cutoffs and were extracted. Finally, ten candidate lncRNAs with log2 fold enrichment scores >1.36, corresponding to >2.5-fold increase in the 3xFN-Tel-TAL sample compared with the 3xFNLDD sample, were identified (Table 2).

### Probes for RNA-FISH

The sequence of the human *SNORD17* probe (synthesized by Life Technologies Inc.) was as follows: 5'-GTGAAATGATGATTCAGTTTATCCATTCGCTGAGTGCGCTGCACTGACCTTCTTCCAAGCCTCAGTTCCTGTTCTAGGAACTTGAGGCTATGTAGCCTGAAAATGCCCTGCAGTCTGCAGTGTTCTACTGTGAACTGCTTGTGTGTTGGCAGGCTACCGGTAAGAATGGTTGGTGTCAGCAGGGACGGGGCCCTCTGAGACCCATCTCACAAAGATGAGTGGTGAAAATCTGATCAC-3'. The human *NEAT1* probe was generated by PCR amplification using 293T genomic DNA as template and the primers (hNEAT1_shortprobe_F and hNEAT1_shortprobe_R) described previously [38]. The RNA-FISH probes were generated by PCR using Cy3-labelled dCTP (Amersham).

### Immunofluorescence combined with RNA-FISH

Immnofluorescence combined with RNA-FISH was performed as described previously [39]. Briefly, cells grown on coverslips were washed with phosphate-buffered saline (PBS), permeabilized with PBS supplemented with 0.5% Triton X-100 for 2 min, and then washed with PBS. Subsequently, cell were fixed for 10 min in 4% paraformaldehyde in PBS, and then permeabilized again with PBS containing 0.5% Triton X-100 for 2 min. After a further wash with PBS, the coverslips were incubated with blocking solution [PBS, 3% (w/v) bovine serum albumin, 0.1% Tween-20, 0.3 μg/μl tRNA (Life Technologies), and 100 units/ml RNasin (Takara Bio Inc)] for 60 min. The cells were then incubated with an anti-TRF2 Ab (Novus Biologicals; NB100-56506) in blocking solution for 60 min at 37°C, and washed twice with PBS containing 0.05% Tween-20. Subsequently, the cells were incubated with AlexaFluor 488-conjugated goat anti-mouse IgG (H+L) (Life Technologies) in blocking solution for 60 min at 37°C, and then washed three times with PBS containing 0.05% Tween-20. After re-fixation with PBS containing 4% paraformaldehyde, the cells were dehydrated sequentially with 70%, 80%, 95%, and 100% ethanol, air-dried, and then hybridized with the RNA probes in hybridization buffer (2xSSC and 50% formamide) at 37°C overnight. After the incubation, the cells were washed three times with hybridization buffer at 37°C, three times with 2xSSC at 37°C, once with 1xSSC at 37°C, once with 4xSSC at room temperature, once with 4xSSC containing 0.1% Tween-20 at room temperature, and once with 4xSSC at room temperature. The signals were visualized using the BZ-9000 fluorescent microscope (Keyence).

## Supporting Information

S1 Table Non-coding RNAs associated with telomeres, as identified by enChIP-RNA-Seq analyses. (XLSX)

S2 Table RNAs detected by enChIP-RNA-Seq analyses. (XLSX)

S3 Table The lncRNAs identified as enriched in telomeres. (XLSX)
